# Effect of Textured Plant Protein Granulation and Presence of Dried Plant Ingredients on Physicochemical Properties of Soy-Based Burger

**DOI:** 10.3390/molecules31050912

**Published:** 2026-03-09

**Authors:** Klaudia Kołodziejczak, Klara Żbik, Iwona Wojtasik-Kalinowska, Anna Onopiuk, Andrzej Poltorak

**Affiliations:** Department of Technique and Food Development, Institute of Human Nutrition Sciences, Warsaw University of Life Sciences, Nowoursynowska 159c Street, 32, 02-776 Warsaw, Poland; klara_zbik@sggw.edu.pl (K.Ż.); anna_onopiuk@sggw.edu.pl (A.O.)

**Keywords:** plant-based, chemical properties, bioactive compounds, food processing, oxidative stability, functional ingredients

## Abstract

Meat analogues have gained high interest from consumers, food producers and the scientific community due to their potential to be a sustainable alternative to traditional meat products. This study investigated the effect of textured soy protein granulation on the structural and biochemical properties of plant-based burgers. Additionally, the incorporation of bioactive plant ingredients, including pomegranate, cardamom, juniper, and carrot powders, was examined for their influence on antioxidant capacity and lipid oxidation during storage. The results demonstrated that larger protein granulation (4–6 mm) enhanced burger hardness and springiness and increased cooking-induced weight loss. The addition of plant-derived bioactive ingredients improved oxidative stability and functional properties, indicating their potential role in extending shelf life and improving product quality. This research provides important knowledge on the role of product formulation and the use of bioactive ingredients in the development of high-quality meat analogues with acceptable physic-chemical properties.

## 1. Introduction

A variety of factors influence consumers’ decisions to reduce their consumption of animal products, but many of these are unrelated to the sensory qualities of these products [1,2,3,4,5]. A significant percentage of consumers are looking for taste sensations similar to animal products after changing their diet. This is the rationale for developing plant-based products with taste, texture and nutritional value comparable to meat or dairy products [5,6,7]. An example of such products is meat analogues. This is a diverse product group that is attracting interest from consumers, producers, and the research community.

A key ingredient in the development of meat analogues is protein. Due to its essential role in building product texture and its wide availability, the most commonly used protein is textured plant protein [8,9,10,11]. There is a wide variety of ingredients in the formulation of meat analogues. This is because replicating the texture of meat and meat products is challenging, and the need to give the product its characteristic color and flavor [6,12]. There is also often a need to mask the taste of the raw materials used, e.g., the aftertaste of soybeans [13,14]. Meat analogues produced from textured plant protein often require significant amounts of water and fat to achieve the desired level of juiciness. Various plant proteins can be used to develop meat analogues. Commercially available plant proteins vary in terms of raw material (e.g., soy, peas, broad beans, rapeseed, lupin), particle shape and size, and technological properties [15,16,17,18].

A wide range of flavorings, additives, and proteins allows the development of ever better meat analogues. These products are now relatively well known to consumers, who are familiar with their presence on grocery shelves. Initially, meat analogues were an exciting novelty for consumers and were enthusiastically purchased. To maintain consumer interest, two main product development strategies are used. The first is improving the texture and taste of the product. The second is introducing new solutions to encourage consumer purchases. This approach aligns with existing food market trends, including the use of ‘clean labels’ and ingredients identified by consumers as health-promoting, such as those belonging to the ‘superfoods’ group [19,20,21].

The available scientific literature indicates numerous directions of research into meat analogues pursued by researchers. To date, studies have focused on consumer attitudes towards meat analogues [22], ecological aspects [23], product life cycle [24], but also technological aspects such as: the impact of process parameters on the quality of textured vegetable protein [7], the use of various additives [25,26], a comparison of the quality and parameters of plant-based analogues with their meat counterparts [27], as well as aspects directly related to the origin and properties of vegetable protein [28]. Nevertheless, multiple published studies focused on limited factors (e.g., particle size or extrusion parameters) or used simplified models that may not fully reflect the actual plant-based burger recipes. This research addresses this gap by evaluating the combined effect of textured soy protein granulation and the addition of bioactive plant powders (pomegranate, cardamom, juniper and carrot) on the chemical and physical properties of soy-based burgers during storage. This study investigated the effect of the granulation of textured plant protein on the physicochemical parameters of soy burgers. Selected dried plant proteins were also incorporated in the formulation of meat analogues in response to current trends and consumer preferences. Dried plant powders are an interesting group of ingredients due to their content of phenolic compounds, natural colorants and essential oils. These ingredients can improve oxidative stability, reduce lipid oxidation, and affect the product’s color and flavor [26,29]. At the same time, their use corresponds with current clean-label trends and consumer interest in naturally derived additives. Pomegranate, cardamom, juniper, and carrot powders were chosen based on scientific evidence and preliminary formulation trials. Pomegranate is a rich source of polyphenols with high antioxidant activity [30,31]. Cardamom and juniper have been proven to contain bioactive compounds that can improve the oxidative stability of food products while also positively affecting their sensory profile [32,33]. The presence of carrot powder in the test recipe is primarily due to its carotenoid content, which can modify color and provide additional nutritional benefits [34]. The final concentrations were selected following pilot experiments to achieve noticeable functional effects—such as increased total phenolic content and enhanced oxidative stability—without reducing sensory quality. Polyphenol and flavonoid content [mg/100g] and DPPH Radical Scavenging Activity [%] of samples of dried plant powders were presented in Table S1.

Certain parameters were investigated during refrigerated storage of the products to enhance understanding of the impact of recipe modifications on the quality of the plant-based burger products. This integrated approach provides a better understanding of how product structure and bioactive ingredients influence key parameters of plant-based meat analogues. To our knowledge, this is the first study to analyze these combined factors in plant-based burgers, providing new insights into product formulation and potential strategies to improve both quality and consumer acceptance.

## 2. Results and Discussion

### 2.1. Color Measurement

The burgers prepared on the soy protein base were subjected to instrumental color analysis (Table 1). During storage, an increase in the brightness (L*) parameter was observed compared to day one in all groups except for the control group and the G1 group with cardamon. No significant differences were observed between the samples prepared with lower granularity proteins until day fourteen, where the highest brightness (46.59) was observed in the samples with carrot powder. In contrast, the lowest brightness was recorded for the samples with pomegranate powder (42.43). The opposite situation was observed for samples with higher granulation protein, where differences (*p* ≤ 0.05) between groups were only observed on days 1 and 7. The burgers analyzed by Botella-Martínez et al. [35] were prepared using liquid beetroot juice. The present study used powdered beetroot juice dissolved in water during sample preparation. The L* parameter in the study by Botella-Martínez et al. [35] ranged from 33.03 to 34.87, a slightly lower value than in the present study, which could be due to the different proportion of the coloring substance in the recipes used. Furthermore, in the present study, carob powder was also used in the burger recipe, which has a darker color and may have contributed to lower brightness of the samples. This combination of at least two coloring ingredients was identified in a study by Ryu et al. [26] as the most effective to achieve the appropriate color in meat analogues. In the study by Ryu et al. [26], beetroot juice and cocoa were used, while in the present study, it was beetroot juice and carob. The brightening of the color of the burgers during storage was related to the use of beetroot juice as one of the color-giving ingredients. This finding is consistent with the results of Kayın et al. [36], who reported a significant increase in the value of the L* parameter during storage of beetroot juice.

Considering the parameter a* (−a* green, +a* red) and the parameter b* (−b* blue, +b* yellow), no significant differences were observed between samples with granulation 1 on day 1 and samples with granulation 2 on day 14 (*p* > 0.05). The samples with pomegranate powder had the highest value of the a* parameter on day 1 (8.80) for granulation1. On day seven, the highest values of this parameter were characterized by samples with carrot powder (8.25) for granulation1 and samples belonging to the control group (10.47) for granulation2. Among samples with lower granulation, the highest b* values were recorded on days 7 and 14 for samples with carrot powder (D7 = 20.39; D14 = 20.43). Among the samples with higher granulation protein, the highest values of the b* parameter were observed on day one for the samples with cardamom powder (18.35) and on day seven for the control group (20.22). The protein granulation and the storage of the burgers did not clearly and significantly affect the a* and b* values in most of the tested samples. The a* value in the tested burgers was comparable to the values obtained by Botella-Martínez et al. [35] for samples with commercial beetroot juice (7.88 and 7.92). For the b* parameter, the results obtained by Botella-Martínez et al. [35] showed a significant difference (8.14–9.72), possibly due to the seasonings used and the color of the textured soy protein. The results of the study by Vu et al. [37] which included instrumental color analysis of commercial plant-based burgers from Impossible Foods (Redwood City, CA, USA) after baking, indicate comparable values for the L* and a* parameters. Regarding the b* parameter, this study obtained significantly higher results, which may be due to differences in the ingredient lists. The Impossible Burger contains different spices and is also enriched with vitamins.

### 2.2. Texture Measurement and Weight Loss [%]

The instrumental analysis of the texture parameters (Table 2 and Table 3) showed that higher granulation of the textured plant protein results in greater springiness in the tested plant-based burgers. Statistically significant differences (*p* ≤ 0.05) were observed for samples with pomegranate powder on days 1 and 14, for the control group on day 14, and for all tested groups on day 7. Storage of the burgers led to an increase in springiness between days 1 and 7, followed by a decrease in the value in this parameter between days 7 and 14, which was observed in most of the tested samples, particularly for granulation two.

On day 1, no statistically significant (*p* > 0.05) differences were observed between groups (control, cardamom, pomegranate, juniper, carrot) and granulations (G1, G2) in terms of the cohesiveness of the burger samples (0.12–0.18). On Day 7, higher springiness was recorded for the higher granulation protein-based samples, except for the juniper powder group. Similar to springiness, most burger samples showed an initial increase in cohesiveness on day 7, followed by a decrease on day 14. However, no statistically significant changes (*p* > 0.05) were observed during storage in samples with G1 protein from the control, cardamom-containing and carrot-containing groups. In a study by Botella-Martínez et al. [35], texture parameters were analyzed in plant burgers prepared with textured soy protein, where significantly lower springiness values (0.10–0.12) were observed compared to the present study, while higher cohesiveness values (0.44–0.53) were recorded in their samples. The differences in these parameters may be primarily due to the formulation composition (despite the similar proportion of textured soy protein—21 and 22%) and the functional properties of the ingredients themselves. In a study by Peñaranda & Garrido [38], the cohesiveness of the tested soy burgers was also significantly higher (0.34–0.38). However, the study used a different thickener (methylcellulose) and a higher content of textured protein.

On the three test days, samples prepared using textured plant protein of higher granulation had a statistically significantly higher value for the hardness parameter than samples based on lower granulation. On day 1, no statistical differences (*p* > 0.05) were observed within the G1 granulation samples concerning the hardness of the burgers (50.08–54.51 N). On the following day (D7), only the control group (114.92 N) was distinguished from the other samples by a higher hardness (*p* ≤ 0.05). A slightly higher variation in hardness was observed among the samples with G2 granulation protein. The samples from the control group had the highest value for this parameter on day 1 (93.85). In all tested groups, regardless of the protein granulation used, storage for 7 days resulted in a statistically significant (*p* ≤ 0.05) increase in the hardness of the burgers. Further storage led to continued increases in hardness in most of the groups, resulting in nearly a twofold increase in this parameter due to water loss.

The soy burgers tested by Botella-Martínez et al. [35] had hardness levels of 22.38–33.30 N, significantly lower than the samples tested in this study. The difference could primarily be attributed to the significantly lower fiber content and the method and duration of heat treatment. In the present study, 2.60% fiber was used, while in the study by Botella-Martínez et al. [35], the proportion of fiber was 1.27%. The burger recipe in this study also included 1.7% potato starch and 4.5% soy protein, which may have contributed to the increased hardness of the burgers. In the study by Jung et al. [39], the hardness values (51.07–91.07 N) were similar to those obtained in the present study, depending on the heat treatment time and temperature. In contrast, the hardness of the burgers in the study by Peñaranda & Garrido [38] was significantly higher, (195.87–247.94 N), probably due to the recipe’s high protein and methylcellulose content. An interesting study was conducted by Lee et al. [27], in which the textural properties of cooked commercial beef burgers and two plant-based burgers—one from the USA and one from South Korea—were examined and compared. About the results of the study by Lee et al. [27], the texture parameter results obtained in this study were significantly higher than those of commercial burgers from the USA (200% for G1 and 400% G2) and South Korea (125% for G1, 200% for G2). Burgers with larger protein granulation had a hardness similar to that of commercial beef burgers. At the same time, the burgers prepared in this study had approximately 4 times lower elasticity and cohesiveness values than both commercial beef and plant-based burgers. This indicates the need to modify the burger recipe in future studies, particularly regarding texturizing agents.

The weight losses (Table 4) due to the heat treatment of the burgers that were observed for each group were not statistically significantly different (*p* > 0.05). Nevertheless, using two granulations of textured soy protein in the plant-based burgers revealed that the higher granulation resulted in greater weight loss in each test group. However, the weight loss difference was statistically significant for the control group and the group containing carrot powder (*p* ≤ 0.05). Weight loss during heat treatment ranged from 11.62 to 12.58% for granulation 1 and 12.77 to 14.09% for granulation 2. Vu et al. [40] reported a higher weight loss (approximately 15%) of soy burgers (Impossible Foods Burger Patties) during baking for 12 min. Also, in the study by Botella-Martínez et al. [35], despite a shorter cooking time, weight loss of 14–17% was observed, which was higher than the present study. This is likely due to differences in formulation and the properties of the selected ingredients, such as fiber, protein powder, textured plant protein, and thickeners.

Differences observed in texture parameters and weight loss between samples can be explained by structural and hydration-related mechanisms associated with particle size in extruded plant protein. Particle size determines the specific surface area available for water absorption and protein interactions. Smaller particles have a higher surface area-to-volume ratio, which promotes more uniform hydration and thus reduces the hardness parameter. In contrast, larger textured protein particles behave more like separate structural units deposited in the matrix [28]. During mechanical testing, these particles can increase physical resistance and, consequently, the hardness parameter. Comparable mechanisms were described by Zhang et al. [41], who showed that increasing the size of TVP particles significantly increased the cutting work, probably due to the mechanical resistance of individual particles. However, the effect of particle size on cooking loss is limited and less consistent, as indicated by Zhang et al. [41], suggesting that raw material properties and hydration level play a more important role than particle size alone.

Storage-related changes in texture can be explained by water redistribution and reorganization of the protein matrix [42]. During refrigerated storage, partial migration of water may occur from the interior of hydrated particles toward the surrounding continuous phase. At the same time, limited surface evaporation and progressive binding of water by hydrocolloids or other water-binding components in the composition can reduce the amount of free water. This change can lead to increased hardness over time.

The effects of dried plant powders on a product formulation’s physical parameters can be explained by several interacting mechanisms. Firstly, the presence of dietary fiber and non-starch polysaccharides in plant powders can increase water-binding capacity and modify the product’s textural properties. Secondly, finely ground plant particles can fill gaps in the protein matrix, altering the microstructure and potentially changing the texture parameters. Protein–polyphenol and protein–polysaccharide interactions during storage may further modify matrix compactness and water immobilization. Nevertheless, these mechanisms are complex and challenging to analyze in a formulation as intricate as the one studied in this research, due to the numerous interactions between raw materials and variations in the chemical composition of plant powders, as well as potential storage-related factors such as heterogeneous moisture loss and microbiological activity that may additionally influence structural stability over time.

### 2.3. Determination of Phenolic Compounds

The prepared soy burgers were analyzed for their polyphenol and flavonoid content (Table 4). The samples with the addition of pomegranate powder (735.44–839.14 mg) exhibited the highest polyphenol content, regardless of the testing day or the granulation of the textured protein. The only exception was on day fourteen, when the highest polyphenol levels were recorded in the samples containing cardamom fruit powder (741.23 mg) in the higher protein granulation group. Among all samples, on each test day, in both protein granulations, the samples belonging to the control group had the lowest polyphenol content (257.31–284.90 mg). Refrigerated storage of the prepared burgers did not have a statistically significant effect (*p* > 0.05) on polyphenol content in most of the samples tested. There was a reduction in polyphenol content in the first 7 days of storage of samples belonging to the control group with smaller granulation and samples with carrot powder and larger granulation. Furthermore, no statistically significant differences (*p* > 0.05) were observed between samples from the same group, tested on the same day, but containing proteins with different granulations.

Similar to the polyphenol content, the highest flavonoid levels were recorded in the samples with pomegranate powder on each test day, regardless of granulation, ranging from 317.39 to 335.36 mg. In contrast, the lowest flavonoid content was recorded in the control group (193.10–214.79 mg). Of the samples formulated with dried plant material, those containing dried carrots exhibited the lowest flavonoid content (238.13–253.8). There were no statistical differences (*p* > 0.05) between the results for the samples in the group containing cardamom and juniper powder. During storage, there was a statistically significant decrease (*p* ≤ 0.05) in flavonoid content between 7 and 14 days only in samples from the control group (granulation 1 and granulation 2). The degree of granulation had no statistically significant effect on the flavonoid content of the soy burgers.

### 2.4. DPPH Radical Scavenging Activity

A plant-based meat analogue based on textured soy protein was analyzed for antioxidant capacity (Table 5). The highest antioxidant capacity was in samples with cardamom fruit powder and pomegranate powder, which did not show statistically significant differences (*p* > 0.05). The lowest values were recorded for the control group samples throughout the study, regardless of the protein granulation used (40.68–47.83%). As with flavonoids, the lowest antioxidant activity values among samples containing plant powders were observed in the carrot powder group (55.48–61.11%). Most of the test groups exhibited the highest DPPH radical scavenging capacity on day one; the exceptions were the groups containing cardamom and pomegranate powder. During storage, a significant decrease (*p* ≤ 0.05) in antioxidant capacity was observed in samples from the control group and those containing carrot powder in the lower granulation protein-based burgers.

Several studies have explored the use of plant ingredients in meat analogues and meat products: [40,43,44,45,46,47]. In the present study, ingredients known for their health-promoting compounds, such as polyphenols and flavonoids, were included in the plant burger recipe: cardamom fruit [48], juniper fruit [49], pomegranate fruit [50], carrot root [51]. Therefore, the results are consistent with the existing scientific literature, which demonstrates that adding plant ingredients containing polyphenols and flavonoids to formulations results in their presence in the finished product, while also influencing its antioxidant capacity.

The observed decrease in antioxidant capacity during refrigerated storage can be explained by the development of lipid oxidation and the degradation of phenolic compounds in plant powders. Phenolic compounds act as radical scavengers, donating hydrogen atoms or electrons, thereby interrupting the crucial phases of lipid oxidation. Over time, these compounds are gradually depleted, which explains the decrease in measured antioxidant activity.

Interactions between proteins and polyphenols may partially protect bioactive compounds by limiting their mobility and reducing oxygen availability. Furthermore, the presence of dietary fiber and polysaccharides in plant powders may change water retention and contribute to texture hardening. This can indirectly slow the diffusion of oxygen, thereby mitigating oxidative reactions. As a result, samples containing plant powders showed slower decrease in antioxidant capacity than the control preparation.

### 2.5. Lipid Oxidation Analysis (TBARS)

The samples stored for 7 and 14 days were subjected to lipid oxidation analysis (Table 5). On each test day, the MDA content was highest in samples belonging to the control group (0.40–1.02 mg). Among the samples with dried plants, the highest content of this compound was present in burgers with carrot powder (0.35–0.81 mg). On day 1, samples containing dried pomegranate, juniper, or cardamom did not show statistically significant differences (*p* > 0.05) (G1 = 0.24–0.28 mg; G2 = 0.21–0.25 mg). On day 7, the lowest values were recorded for samples with cardamom and pomegranate, which were statistically significantly different (*p* ≤ 0.05) from samples with juniper. In contrast, pomegranate powder samples showed lower values on day 14 (G1 = 0.42 mg, G2 = 0.39 mg Throughout the storage period, the MDA content increased significantly (*p* ≤ 0.05) in all samples, reaching its peak on day 14.). The exceptions were samples with pomegranate and lower granulation protein and juniper and higher granulation protein, where the MDA content did not increase from day 7, and samples with pomegranate and higher granulation protein, where the change occurred only between days 7 and 14. The granulation of the textured protein did not significantly affect lipid oxidation (*p* > 0.05) in most of the samples, except in the control group on day 14, where the MDA content was significantly higher in G1 granulation samples.

Ariz et al. [52] demonstrated that a heat-treated plant-based (pea) protein burger had an MDA content of 0.42 ppm, which is comparable to the results obtained in the present study for the control group on day one. This finding aligns with research on the potential of plant-based ingredients in meat products and meat analogues to reduce lipid oxidation. The incorporation of plant-based ingredients has been shown to reduce lipid oxidation during storage and thermal processing of products [53,54].

### 2.6. Semi-Consumer Analysis of Plant-Based Burgers

The plant-based burger samples were subjected to a semi-consumer analysis. The results of the evaluation of selected sensory attributes such as color, smell, taste, texture, and overall acceptability are presented in Table 6. Burgers containing pomegranate powder received the highest scores for color, aroma, taste, and overall acceptability, regardless of the granulation tested. In terms of texture, samples with carrot powder and juniper received the highest ratings in granulation 1, while in granulation 2, the control group burgers were rated as having the best texture by the evaluators. The samples with cardamom and juniper, and the control group, received the lowest ratings for all parameters.

The semi-consumer evaluation showed differences in the acceptance of the tested plant-based burger variants depending on the plant powder additive used. Differences in perception of texture and color largely corresponded to the results of the instrumental TPA analysis and color parameter measurements (L*, a*, b*). In the color assessment, the variants with the highest L* values in burgers with granulation 1 were perceived as more attractive. At the same time, significantly lower L* values in samples with protein granulation 2 resulted in a lower color rating. The addition of powders rich in natural colorants (e.g., carotenoids in carrots) increased the a* or b* parameter values, resulting in a warmer product shade and, in some cases, an improvement in visual assessment. Samples with the lowest a* parameter values were rated worst for color and overall acceptability. The texture assessment results also corresponded to the semi-consumer assessment results. Samples with higher hardness in instrumental analysis received higher scores in the texture and overall acceptability assessment. Samples with the lowest hardness were rated significantly lower.

## 3. Materials and Methods

### 3.1. Sample Preparation and Baking Procedure

**Figure 1:**
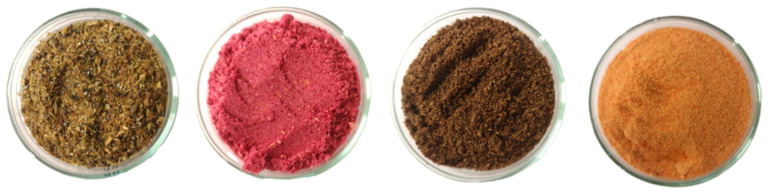
Dried plant powders. From left cardamom fruit, pomegranate fruit, juniper fruit, and carrot.

The following raw materials were used to prepare plant-based soy protein burgers: textured soy protein in two granulations: 2–4 (G1) and 4–6 mm (G2) (Sojaprotein, Bečej, Serbia), red beet juice powder (Planteon, Bydgoszcz, Poland), carob powder (Planteon, Bydgoszcz, Poland), rapeseed oil (Złote Łany, Bielsko-Biała, Poland), wheat fiber (Promienie słońca, Poland), pea fiber (Promienie słońca, Rzgów, Poland), soy protein isolate (Libra, Olsztyn, Poland), potato starch (PPZ Trzemeszno Sp. z o.o., Trzemeszno, Poland), dried onion (Planteon, Bydgoszcz, Poland), amidated pectin (Rafex, Kąty Wrocławskie, Poland), xanthan gum (Rafex, Kąty Wrocławskie, Poland), beef seasoning (salt, mustard, corn, sweet paprika, allspice, hot paprika, granulated garlic, sugar, marjoram, caraway, thyme, bay leaf) (Planteon, Bydgoszcz, Poland), salt (Solino, Inowrocław, Poland), dried cardamom fruit (Green Essence, Warsaw, Poland), dried pomegranate (AGPol, Motycz, Poland), dried juniper fruit (Zioła z Kurpi, Kadzidło, Poland), dried carrot (Planteon, Bydgoszcz, Poland). Dried plant powders (cardamom fruit, pomegranate fruit, juniper fruit, and carrot) are presented on Figure 1.

The composition of plant-based meat substitutes is presented in Table 7 and was developed based on preliminary research conducted during the design of the experiment described in this article. For additives such as pectins and xanthan gum, the limits specified in Regulation (EC) No. 1333/2008 on food additives were applied.

A meat analogue in the form of a soy protein-based burger was prepared by hydrating the textured protein with a mixture of water, beet juice powder, and carob. The other ingredients were then blended thoroughly, resulting in a homogeneous mixture. The textured plant protein and the prepared mixture were combined through careful mixing. Using a burger press, burgers with a uniform diameter of 10 cm and a thickness of 1.5 cm were formed. Ten burgers were prepared for each research group. The burgers were weighed and then heat-treated in a combi oven (Küppersbusch, Gelsenkirchen, Germany) until the geometric center of the product reached 80 °C (about 12 min). After cooling, the burgers were weighed. Samples were vacuum-packaged using a chamber vacuum sealer (Edesa VAC-20 SL 2A, Edesa, Basauri, Spain) at a controlled 50% vacuum level. Samples were sealed in smooth, three-side-sealed vacuum pouches (150 × 200 mm) made of multilayer PA/PE (polyamide/polyethylene) film with a nominal thickness of 70 μm. The film exhibited an oxygen transmission rate (OTR) of 65 cm^3^·m^−2^·24 h^−1^·bar^−1^ (ASTM D3985, 23 °C/0% RH) and a water vapor transmission rate (WVTR) of 9 g·m^−2^·24 h^−1^ (ASTM F1249, 38 °C/90% RH) under standard test conditions. Packed samples were stored in 4 °C for further testing.

The burger samples were analyzed for color, texture, TPC, TFC, antioxidant activity, and lipid oxidation level on days 1, 7, and 14 of storage. The analysis time points were selected based on typical experimental designs for analyzing the properties of plant-based meat analogues during refrigerated storage and on market data on the average shelf-life of commercial plant-based burgers. Furthermore, the samples were subjected to weight-loss and semi-consumer analyses on the first day of storage.

### 3.2. Color Measurement

A color analysis of the burgers based on the CIE Lab* system using a Minolta CR-400 colorimeter (Konica Minolta Inc., Tokyo, Japan), using the reflectance method based on Szpicer et al. [55]. Before using the device, it was calibrated using a white standard with values of L* = 98.45, a* = −0.10 and b* = −0.13. Three parameters were examined: L* (lightness, L* = 0/black and L* = 100/white), a* (−a* green, +a* red) and b* (−b* blue, +b* yellow). The arithmetic average of the ten measurements was calculated for each parameter.

### 3.3. Texture Measurement

The texture characteristics of the meat analogues were measured, including springiness (the ratio of the height reached after the first compression to the primary sample height), cohesiveness (the proportion of the area under the curve from the second to the first compression) and hardness (the maximum force of the first compression; N/cm^2^). An Instron 5965 universal testing machine (Instron, Norwood, MA, USA) was used. A double compression test was performed by compressing the sample to 50% of its initial height. The relaxation time was 50 milliseconds, the compression force was 0.04 N, and the head speed was 10 mm/min. Measurements were made on cylindrical samples with a height of 1.5 cm and a diameter of 2.5 cm, with each measurement repeated six times.

### 3.4. Weight Loss

A precise control of the weight of the plant-based burgers was carried out, which made it possible to calculate the weight loss percentage. The percentage weight loss was calculated according to Equation (1).

(1) $$WL=\frac{W_{R}-W_{H}}{W_{R}}*100\%,$$

WL represents the percentage weight loss after heat treatment [%], W_R_ is the initial weight of the sample, and W_H_ is the weight of the sample after heat treatment.

### 3.5. Determination of Phenolic Compounds

#### 3.5.1. Total Phenolic Content (TPC)

Total phenolic compound (TPC) content was determined according to the method described by Singleton and Rossi [56]. In the first step, 2.5 g of the sample was homogenized using an Ultra Turrax device (IKA T18 basic, Staufen, Germany) with 7.5 mL of ethanol. The extract was subsequently filtered through filter paper. The 0.1 mL of filtrate was mixed with 6.0 mL of distilled water and 0.5 mL of Folin and Ciocalteu phenol reagent. After that, 1.5 mL of sodium bicarbonate (at a concentration of 7.5% *w*/*v*) was added after 3 min. The mixture was incubated for 40 min in a water bath (WNB 7 Memmert, Schwabach, Germany) set at 40 °C. Spectrophotometric absorbance was measured at λ = 760 nm (Tecan Spark™ 10 M, Männedorf, Switzerland). Total phenolic content was expressed as gallic acid (GA) equivalent based on a standard curve. Results are presented as the arithmetic mean of three replicates in mg GA per 100 g sample.

#### 3.5.2. Total Flavonoid Content (TFC)

The total flavonoid content (TFC) was measured by spectrophotometric analysis based on the Onopiuk et al. [57] methodology. To 1 mL of ethanol extract of the sample (prepared as described in Section 3.5.1), 1 mL of 2% aqueous solution of AlCl_3_-6H_2_O was added and mixed thoroughly. The samples were subsequently incubated at room temperature for 10 min. At the same time, a blank sample was prepared without extract. Absorbance was measured at 420 nm (Tecan Spark™ 10 M, Männedorf, Switzerland). The total flavonoid content was calculated using the standard curve. The results are presented as the arithmetic mean of the three measurements in mg of quercetin per 100 g of sample.

### 3.6. DPPH Radical Scavenging Activity

The total antioxidant activity of heat-treated burgers was analyzed. For this purpose, the DPPH (1,1-diphenyl-2-picrylhydrazyl) radical was used according to the procedure developed by Sánchez-Moreno et al. [58]. At the initial stage of the study, a 2.5 g sample was weighed out and homogenized with 7.5 mL of ethanol for 2 min at 7500 rpm (Ultra Turrax homogeniser, T18 basic, IKA Werke, Staufen, Germany). The samples were then extracted for 10 min at room temperature using a shaker (MyLab SLRM-3, NanoEnTek Inc., Seoul, Republic of Korea). After extraction, the samples were centrifuged at 1800 rpm for 10 min (MPW Med. Instruments, Warsaw, Poland). To 0.5 mL of liquid collected over the sediment, 3.5 mL of 0.1 mM ethanolic DPPH solution was added. The samples were vortexed and then incubated in the dark for 20 min at 25 °C. Absorbance was measured spectrophotometrically at 510 nm (SparkTM 10M, Tecan Group Ltd., Männedorf, Switzerland). Ethanol was used as a control. Measurements were made in triplicate, and the result of total antioxidant activity was presented as the arithmetic mean of the values calculated according to Formula (2):

(2) $$TAA=\frac{1-A_{S}}{A_{C}}*100,\;$$

where TAA—reduction of DPPH [%], A_S_—the absorbance of the test sample and A_C_—the absorbance of the control (containing all the reagents except extract).

### 3.7. Lipid Oxidation Analysis (TBARS)

Soy-based burger samples (5 g) were placed in 50 mL of TCA (trichloroacetic acid) solution (20% TCA solution mixed with 1.6% concentrated phosphoric acid, cooled to 4 °C) by the procedure by Pikul et al. [59]. Then, 2.5 mL of antioxidant (a 1:1 water-ethanol solution containing 0.5% propyl gallate and 0.5% EDTA) was added. After homogenization, the mixture was filtered through the Whatman Class 1 filter paper. The sample volume was increased to 100 mL with a 1:1 mixture of water and ethanol. A 5 mL of the sample mixture was mixed with 5 mL of 0.02 M TBA (thiobarbituric acid) solution. After 40 min of incubation in a boiling water bath (WNB 7 Memmert, Büchenbach, Germany), spectrophotometric was measured at 532 nm standard (control sample—TCA: water mixture). TBARS (thiobarbituric acid reactive substances) concentration was calculated using 1,1,3,3-tetramethoxypropane (TEP, 0–11 μM) as a standard. The TBARS value has been expressed as milligrams of malondialdehyde (MDA) per kilogram of sample (mg MDA/kg sample).

### 3.8. Semi-Consumer Sensory Evaluation

A semi-consumer sensory evaluation of plant-based burgers was conducted under controlled laboratory conditions. The study involved *n* = 35 participants (19 females and 16 males), aged 21–58 years. All participants provided informed consent prior to the evaluation. The assessment was carried out in individual sensory booths [60,61].

#### 3.8.1. Sample Preparation

Burgers were prepared by baking to an internal temperature of 80 °C and served at 50 °C. Portions of burger with dimensions 25 × 25 mm were prepared from each formulation. Samples were coded with random three-digit numbers and presented once to each panelist in randomized order on white, odorless disposable plates. A 5 min interval was maintained between samples to prevent sensory fatigue. Still water and white bread were provided as palate cleansers.

#### 3.8.2. Sensory Assessment

A 9-point hedonic scale was used, where 1 indicated “extremely undesirable” and 9 indicated “extremely desirable.” The following attributes were evaluated: color, aroma, taste, texture, overall acceptability. Results were expressed as numerical values ranging from 1 to 9 points. The order of sample presentation was randomized for each panelist to minimize potential order and carry-over effects.

### 3.9. Statistical Analysis

Each experimental variant consisted of 3–6 independent replicates (biological replicates). All physicochemical determinations were performed in 10–15 technical replicates per sample. The experiment was conducted using a completely randomized design. Prior to ANOVA, the normality of data distribution was verified using the Shapiro–Wilk test, and homogeneity of variance was assessed. Data were analyzed using one-way analysis of variance (ANOVA), and post hoc comparisons were performed using Tukey’s RIR test at a significance level of *p* < 0.05. Statistical analyses were carried out using Statistica version 13.1 (TIBCO Software Inc., Tulsa, OK, USA).

## 4. Limitations and Future Perspectives

This study provided information on the impact of textured soy protein granulation and the addition of bioactive plant powders on the quality properties of plant-based burgers, but several limitations should be noted. Firstly, only two protein granulations from a single supplier were analyzed, and detailed parameters of the texturization process were not available. This limits the ability to assess the impact of technological conditions and raw material variability on the product’s functional properties. It is well known that the properties of protein depend, among other things, on its molecular structure, which is fundamentally related to the extrusion process and the subsequent parameters of the extruded protein. Secondly, the study was limited to the impact of storage time on physicochemical parameters. It did not include microbiological analysis, which limits the conclusions that can be drawn about the product’s safety during storage. The nutritional aspects of the product, including analyses of nutritional value and digestibility, were also omitted, preventing a comprehensive assessment of the nutritional quality of the burgers developed. In addition, storage was limited in time and conducted under specific conditions that do not fully reflect the diverse market realities and post-purchase treatment.

The limitations of this study point to future research directions that could provide valuable insights into meat analogues. In future studies, it would be reasonable to consider a broader range of plant protein sources, such as pea, wheat, faba bean or lupin protein, as well as protein blends, in order to more accurately determine the effect of protein type on texture, water holding capacity, oxidative stability and other properties of the product. It would also be helpful to examine a broader range of granulations and their mixtures, as well as to compare raw materials from different manufacturers or those produced under different extrusion conditions, to understand structure-function relationships better. The research should be supplemented by microstructural analysis (e.g., light microscopy, SEM, or 3D imaging techniques), enabling direct assessment of the organization and porosity of the protein matrix and linking these to the results of instrumental texture and water holding capacity analyses. It is also worth extending the scope of storage studies to longer periods, varying temperature conditions, and packaging, including microbiological assessment, to enable more accurate modeling of product shelf life. Further studies should also include a detailed analysis of nutritional value and digestibility to assess the actual nutritional value and market potential of the plant-based burgers developed. The implementation of the indicated research directions will contribute to a deeper understanding of the functional properties of meat analogues and enable the development of products with high quality, stability and consumer acceptability.

## 5. Conclusions

The high consumer interest in meat analogues highlights the need for extensive research in this area. The most commonly purchased meat analogues are burger-shaped products. With soy protein being the most widely used ingredient. Research conducted on soy protein-based burgers have demonstrated the influence of variables such as the granulation of textured plant protein, incorporation of dried vegetable ingredients, and storage duration on the physicochemical properties of the final product. It was observed that using a higher granulation of plant protein (4–6 mm) increases the springiness and hardness of the product, whereas finer granulation (2–4 mm) results in lower weight loss during thermal processing. With increasing storage time, a brightening of the color of the burgers and an increase in their hardness were observed, reflecting expected changes in the values of these parameters. Furthermore, the addition of dried plant-derived ingredients, documented to contain polyphenols and flavonoids, enhanced the antioxidant capacity of the meat analogue and reduced MDA content, compared to the control group, throughout the storage period studied. Therefore, the selection of ingredients and their forms significantly influences the physicochemical properties of the final product, potentially leading to their improvement.

## Figures and Tables

**Table 1:** CIE parameters (L*, a* and b*) in baked plant-based meat analogues.

Group	L*			a*			b*		
	D1	D7	D14	D1	D7	D14	D1	D7	D14
Granulation 1 (2–4 mm)									
Control	43.70 ± 1.92	45.02 ± 1.12 **	44.05 ± 0.8 ^B^	7.63 ± 1.03	7.54 ± 0.4 ^BC^*	7.64 ± 0.43 ^B^	18.02 ± 1.59	18.6 ± 0.67 ^A^*	18.38 ± 0.53 ^B^
Cardamon	43.09 ± 1.56	44.52 ± 0.79	44.05 ± 1.46 ^B^*	7.29 ± 0.76 ^b^	6.50 ± 0.41 ^Aa^	6.77 ± 5.43 ^Aab^	18.2 ± 1.34 ^b^	17.21 ± 0.78 ^Bb^	16.94 ± 0.46 ^Ca^
Pomegranate	42.17 ± 0.95 ^a^	44.68 ± 1.07 ^b^	42.43 ± 0.97 ^Aa^*	7.60 ± 0.77 *	7.26 ± 0.71 ^AB^	7.24 ± 6.43 ^AB^	17.51 ± 1.38 ^b^	17.63 ± 1.44 ^ABb^	15.57 ± 0.71 ^Aa^
Juniper	42.76 ± 1.35 ^a^	44.35 ± 0.6 ^b^	44.00 ± 1.08 ^Bb^	7.17 ± 0.8	6.57 ± 0.35 ^A^	6.98 ± 3.43 ^A^	17.66 ± 1.36	17.12 ± 0.76 ^B^	17.48 ± 0.98 ^C^
Carrot	43.16 ± 1.55 ^a^	44.77 ± 0.8 ^b^	46.59 ± 0.27 ^Cc^	7.62 ± 0.65	8.25 ± 1.36 ^C^**	8.42 ± 7.43 ^C^**	18.66 ± 1.19 ^a^	20.39 ± 0.83 ^Cb^**	20.43 ± 0.16 ^Db^**
Granulation 2 (4–6 mm)									
Control	42.72 ± 1.6 ^ABa^	43.23 ± 0.71 ^Aa^*	45.56 ± 1.34 ^b^	7.39 ± 0.74 ^Aa^	10.47 ± 0.93 ^Cb^**	7.00 ± 2.43 ^a^	17.18 ± 0.83 ^Aa^	20.22 ± 0.54 ^Cb^**	17.42 ± 0.8 ^a^
Cardamon	43.91 ± 1.5 ^Ba^	43.89 ± 0.73 ^ABa^	46.58 ± 1.98 ^b^**	7.44 ± 0.94 ^A^	6.56 ± 0.29 ^AB^	7.25 ± 8.43	18.35 ± 0.63 ^Bb^	17.44 ± 0.38 ^Ba^	17.95 ± 1.15 ^ab^
Pomegranate	42.91 ± 1.03 ^ABa^	43.55 ± 1.05 ^ABa^	45.73 ± 1.92 ^b^**	8.8 ± 0.96 ^Bb^**	7.16 ± 0.19 ^Ba^	7.3 ± 5.43 ^a^	17.29 ± 0.67 ^Ab^	16.23 ± 0.25 ^Aa^	16.74 ± 1.46 ^ab^
Juniper	41.94 ± 1.52 ^Aa^	45.6 ± 1.32 ^Bb^	44.89 ± 1.32 ^b^	6.96 ± 0.63 ^A^	6.55 ± 0.4 ^A^	7.18 ± 6.43	16.55 ± 1.51 ^Aa^	17.44 ± 0.74 ^Bab^	18.12 ± 1.56 ^b^
Carrot	43.72 ± 1.5 ^ABa^	44.65 ± 0.91 ^Ba^	46.27 ± 1.8 ^b^	7.36 ± 0.54 ^Ab^	6.34 ± 0.08 ^Aa^*	6.94 ± 6.43 ^ab^*	18.14 ± 1.20 ^A^	17.39 ± 0.55 ^B^*	18.36 ± 1.49 *

Values are presented as mean ± standard deviation ^A–D^—averages in columns with different letters indicate statistical differences between samples belonging to different groups (control, cardamom, pomegranate, juniper, carrot) within one day and one granulation; ^a–c^—averages in rows marked with different letters indicate statistical differences between samples belonging to the same group (control, cardamom, pomegranate, juniper, carrot) within the same granulation, but on a different day; *–**—averages in columns with different number of * indicate statistical differences between samples belonging to the same group (control, cardamom, pomegranate, juniper, carrot) on the same day, but with different granulation; *p* ≤ 0.05.

**Table 2:** Springiness and cohesiveness of baked plant-based meat analogues.

Group	Springiness [−]			Cohesiveness [−]		
	D1	D7	D14	D1	D7	D14
Granulation 1 (2–4 mm)						
Control	0.24 ± 0.05 ^B^	0.14 ± 0.05 ^A^*	0.18 ± 0.09 *	0.16 ± 0.03	0.1 ± 0.06 ^AB^*	0.11 ± 0.09
Cardamon	0.21 ± 0.03 ^ABab^	0.15 ± 0.03 ^Aa^*	0.23 ± 0.05 ^b^	0.16 ± 0.02	0.13 ± 0.04 ^AB^*	0.18 ± 0.09
Pomegranate	0.19 ± 0.04 ^ABab^*	0.20 ± 0.04 ^ABb^*	0.14 ± 0.05 ^a^*	0.15 ± 0.04 ^ab^	0.18 ± 0.02 ^BCb^*	0.11 ± 0.09 ^a^*
Juniper	0.20 ± 0.04 ^ABab^	0.27 ± 0.04 ^Bb^*	0.18 ± 0.03 ^a^	0.14 ± 0.04 ^a^	0.21 ± 0.02 ^Cb^	0.14 ± 0.09 ^a^
Carrot	0.17 ± 0.04 ^A^	0.14 ± 0.04 ^A^*	0.22 ± 0.08	0.12 ± 0.03	0.10 ± 0.06 ^A^*	0.16 ± 0.09 *
Granulation 2 (4–6 mm)						
Control	0.22 ± 0.04 ^a^	0.38 ± 0.04 ^ABb^**	0.31 ± 0.07 ^Bb^**	0.15 ± 0.02 ^a^	0.24 ± 0.02 ^ABc^**	0.19 ± 0.09 ^Bb^
Cardamon	0.21 ± 0.05 ^a^	0.35 ± 0.05 ^Ab^**	0.19 ± 0.06 ^Aa^	0.14 ± 0.04 ^a^	0.23 ± 0.01 ^Ab^**	0.12 ± 0.09 ^ABa^
Pomegranate	0.25 ± 0.01 ^a^**	0.42 ± 0.01 ^Bb^**	0.24 ± 0.03 ^ABa^**	0.18 ± 0.01 ^a^	0.25 ± 0.02 ^ABb^**	0.16 ± 0.09 ^Ba^**
Juniper	0.24 ± 0.04 ^a^	0.42 ± 0.04 ^Bb^**	0.24 ± 0.08 ^ABa^	0.17 ± 0.02 ^a^	0.26 ± 0.03 ^Bb^	0.16 ± 0.09 ^Ba^
Carrot	0.22 ± 0.02 ^a^	0.36 ± 0.02 ^ABb^**	0.14 ± 0.05 ^Aa^	0.15 ± 0.01 ^a^	0.24 ± 0.01 ^ABb^**	0.07 ± 0.09 ^Aa^**

Values are presented as mean ± standard deviation ^A–C^—averages in columns with different letters indicate statistical differences between samples belonging to different groups (control, cardamom, pomegranate, juniper, carrot) within one day and one granulation; ^a–c^—averages in rows marked with different letters indicate statistical differences between samples belonging to the same group (control, cardamom, pomegranate, juniper, carrot) within the same granulation, but on a different day; *–**—averages in columns with different number of * indicate statistical differences between samples belonging to the same group (control, cardamom, pomegranate, juniper, carrot) on the same day, but with different granulation; *p* ≤ 0.05.

**Table 3:** Hardness [N] and weight loss [%] of baked plant-based meat analogues.

Group	Hardness [N]			Weight Loss [%]
	D1	D7	D14	D1
Granulation 1 (2–4 mm)				
Control	51.54 ± 3.94 ^a^*	114.92 ± 3.24 ^Bc^*	103.12 ± 4.77 ^Cb^*	12.29 ± 0.01 *
Cardamon	52.63 ± 2.70 ^a^*	94.84 ± 2.39 ^Ab^*	98.95 ± 6.44 ^BCb^*	12.57 ± 0.01
Pomegranate	53.68 ± 3.48 ^a^*	93.23 ± 3.75 ^Ab^*	103.3 ± 4.92 ^Cc^*	12.00 ± 0.01
Juniper	50.08 ± 3.97 ^a^*	86.71 ± 8.35 ^Ab^*	93.29 ± 5.45 ^ABb^*	12.58 ± 0.01
Carrot	54.51 ± 3.23 ^a^*	90.71 ± 4.90 ^Ab^*	88.84 ± 3.88 ^Ab^*	11.62 ± 0.01 *
Granulation 2 (4–6 mm)				
Control	93.85 ± 6.55 ^Ba^**	119.86 ± 9.37 ^Bb^**	133.52 ± 6.32 ^Bc^**	14.08 ± 0.01 **
Cardamon	84.47 ± 9.82 ^ABa^**	123.55 ± 4.83 ^Bb^**	167.95 ± 6.37 ^Cc^**	13.75 ± 0.02
Pomegranate	74.70 ± 4.6 ^Aa^**	108.15 ± 5.37 ^Ab^**	130.20 ± 5.43 ^ABc^**	12.81 ± 0.01
Juniper	81.11 ± 3.27 ^Aa^**	103.89 ± 6.06 ^Ab^**	121.34 ± 6.47 ^Ac^**	12.77 ± 0.01
Carrot	83.50 ± 4.17 ^ABa^**	127.02 ± 5.25 ^Bb^**	160.41 ± 5.44 ^Cc^**	14.09 ± 0.01 **

Values are presented as mean ± standard deviation ^A–C^—averages in columns with different letters indicate statistical differences between samples belonging to different groups (control, cardamom, pomegranate, juniper, carrot) within one day and one granulation; ^a–c^—averages in rows marked with different letters indicate statistical differences between samples belonging to the same group (control, cardamom, pomegranate, juniper, carrot) within the same granulation, but on a different day; *–**—averages in columns with different number of * indicate statistical differences between samples belonging to the same group (control, cardamom, pomegranate, juniper, carrot) on the same day, but with different granulation; *p* ≤ 0.05.

**Table 4:** Polyphenol and flavonoid content [mg/100g] of samples of plant-based meat analogues.

Group	Polyphenols [mg GAE/100 g]			Flavonoids [mg Quercetin/100 g]		
	D1	D7	D14	D1	D7	D14
Granulation 1 (2–4 mm)						
Control	284.90 ± 8.97 ^Ab^	260.25 ± 4.88 ^Aa^	269.19 ± 6.59 ^Aa^	214.79 ± 3.98 ^Ab^	214.44 ± 7.17 ^Ab^	199.10 ± 5.04 ^Aa^
Cardamon	673.76 ± 7.43 ^Da^	665.52 ± 7.71 ^Da^	674.80 ± 6.10 ^Da^	280.94 ± 3.65 ^Ca^	284.24 ± 4.29 ^Ca^	284.46 ± 9.46 ^Ca^
Pomegranate	744.11 ± 8.08 ^Ea^	735.44 ± 4.32 ^Ea^	742.25 ± 10.91 ^Ea^	335.36 ± 5.55 ^Da^	324.44 ± 7.20 ^Da^	334.76 ± 4.39 ^Da^
Juniper	552.65 ± 7.96 ^Ca^	578.13 ± 9.08 ^Cb^	574.05 ± 3.14 ^Cb^	278.43 ± 7.19 ^Ca^	270.05 ± 5.71 ^Ca^	270.27 ± 4.73 ^Ca^
Carrot	408.72 ± 3.33 ^Ba^	399.26 ± 8.93 ^Ba^	413.03 ± 9.53 ^Ba^	245.10 ± 5.28 ^Ba^	238.13 ± 3.03 ^Ba^	244.14 ± 6.12 ^Ba^
Granulation 2 (4–6 mm)						
Control	273.91 ± 9.88 ^Aa^	266.26 ± 5.25 ^Aa^	257.31 ± 9.56 ^Aa^	206.15 ± 3.70 ^Ab^	206.71 ± 2.34 ^Ab^	193.19 ± 6.55 ^Aa^
Cardamon	661.56 ± 8.70 ^Da^	663.94 ± 8.35 ^Da^	674.65 ± 10.71 ^Da^	280.76 ± 4.14 ^Ca^	279.93 ± 7.91 ^Ca^	288.83 ± 6.29 ^Ca^
Pomegranate	839.14 ± 7.45 ^Ea^	736.24 ± 6.09 ^Ea^	741.23 ± 7.85 ^Ea^	326.11 ± 5.17 ^Da^	317.39 ± 11.86 ^Da^	327.94 ± 11.62 ^Da^
Juniper	566.47 ± 6.56 ^Ca^	564.51 ± 3.58 ^Ca^	556.32 ± 10.22 ^Ca^	285.70 ± 2.30 ^Ca^	281.58 ± 4.13 ^Ca^	277.96 ± 3.66 ^Ca^
Carrot	418.74 ± 6.40 ^Bb^	401.68 ± 3.94 ^Ba^	395.71 ± 4.22 ^Ba^	245.02 ± 2.38 ^Ba^	248.08 ± 8.11 ^Ba^	253.81 ± 9.79 ^Ba^

Values are presented as mean ± standard deviation ^A–E^—averages in columns with different letters indicate statistical differences between samples belonging to different groups (control, cardamom, pomegranate, juniper, carrot) within one day and one granulation; ^a,b^—averages in rows marked with different letters indicate statistical differences between samples belonging to the same group (control, cardamom, pomegranate, juniper, carrot) within the same granulation, but on a different day; *p* ≤ 0.05.

**Table 5:** DPPH radical scavenging activity [%] and lipid oxidation TBARS [mg MDA/kg] of samples of plant-based meat analogues.

Group	DPPH Radical Scavenging Activity [%]			Lipid Oxidation TBARS [mg MDA/kg]		
	D1	D7	D14	D1	D7	D14
Granulation 1 (2–4 mm)						
Control	47.83 ± 1.58 ^Ab^	44.44 ± 1.42 ^Ab^	42.77 ± 1.03 ^Aa^	0.48 ± 0.03 ^Ba^	0.73 ± 0.03 ^Cb^	1.02 ± 0.04 ^Dc^**
Cardamon	78.55 ± 0.55 ^Da^	79.47 ± 2.40 ^Da^	77.00 ± 1.37 ^Da^	0.28 ± 0.05 ^Aa^	0.44 ± 0.02 ^Ab^	0.61 ± 0.04 ^Bc^
Pomegranate	76.79 ± 0.81 ^Da^*	79.06 ± 0.59 ^Db^**	79.60 ± 0.57 ^Db^	0.24 ± 0.02 ^Aa^	0.37 ± 0.03 ^Ab^	0.42 ± 0.05 ^Ab^
Juniper	70.65 ± 1.62 ^Ca^	68.64 ± 1.16 ^Ca^	67.34 ± 3.10 ^Ca^	0.28 ± 0.03 ^Aa^	0.55 ± 0.02 ^Bb^	0.63 ± 0.04 ^Bc^
Carrot	61.11 ± 2.42 ^Bb^	59.12 ± 2.07 ^Bb^	56.04 ± 1.43 ^Ba^	0.40 ± 0.04 ^Ba^	0.66 ± 0.04 ^Cb^	0.81 ± 0.04 ^Cc^
Granulation 2 (4–6 mm)						
Control	46.37 ± 0.84 ^Aa^	44.15 ± 1.25 ^Aa^	40.68 ± 3.87 ^Aa^	0.40 ± 0.02 ^Ba^	0.71 ± 0.06 ^Cb^	0.89 ± 0.05 ^Dc^*
Cardamon	77.65 ± 1.70 ^Da^	76.62 ± 2.88 ^Da^	78.14 ± 0.58 ^Da^	0.25 ± 0.02 ^Aa^	0.39 ± 0.03 ^Ab^	0.55 ± 0.05 ^Bc^
Pomegranate	78.91 ± 0.66 ^Db^**	76.12 ± 0.69 ^Da^*	79.53 ± 0.75 ^Db^	0.21 ± 0.04 ^Aa^	0.32 ± 0.02 ^Aa^	0.39 ± 0.04 ^Ab^
Juniper	66.30 ± 2.31 ^Ca^	65.98 ± 1.99 ^Ca^	65.91 ± 2.01 ^Ca^	0.25 ± 0.01 ^Aa^	0.56 ± 0.04 ^Bb^	0.59 ± 0.04 ^Bb^
Carrot	59.54 ± 1.85 ^Ba^	57.26 ± 1.84 ^Ba^	55.48 ± 2.77 ^Ba^	0.35 ± 0.05 ^Ba^	0.59 ± 0.04 ^Bb^	0.72 ± 0.02 ^Cc^

Values are presented as mean ± standard deviation ^A–D^—averages in columns with different letters indicate statistical differences between samples belonging to different groups (control, cardamom, pomegranate, juniper, carrot) within one day and one granulation; ^a–c^—averages in rows marked with different letters indicate statistical differences between samples belonging to the same group (control, cardamom, pomegranate, juniper, carrot) within the same granulation, but on a different day; *–**—averages in columns with different number of * indicate statistical differences between samples belonging to the same group (control, cardamom, pomegranate, juniper, carrot) on the same day, but with different granulation; *p* ≤ 0.05.

**Table 6:** Semi-consumer evaluation of plant-based burgers.

Group	Color	Aroma	Taste	Texture	Overall Acceptability
Control	7.17 ± 0.97	6.83 ± 0.84	7.03 ± 0.84	7.83 ± 0.91	6.71 ± 0.91
Cardamon	6.83 ± 0.97	6.97 ± 0.94	7.09 ± 0.81	7.77 ± 0.83	7.23 ± 0.83
Pomegranate	8.40 ± 0.49	7.69 ± 1.06	7.51 ± 1.18	7.86 ± 0.93	8.06 ± 0.83
Juniper	7.60 ± 1.22	7.26 ± 1.23	7.46 ± 1.15	7.91 ± 0.77	7.91 ± 0.91
Carrot	8.00 ± 0.83	7.11 ± 0.82	7.17 ± 0.84	7.91 ± 0.73	7.29 ± 0.78
Control	7.17 ± 0.97	6.83 ± 0.84	7.03 ± 0.84	7.83 ± 0.91	6.71 ± 0.91
Cardamon	6.91 ± 0.91	6.49 ± 1.05	6.60 ± 1.44	6.40 ± 1.81	6.17 ± 1.38
Pomegranate	7.51 ± 1.11	7.11 ± 0.85	7.03 ± 0.77	6.20 ± 1.82	6.94 ± 0.89
Juniper	7.09 ± 1.70	6.89 ± 0.78	6.46 ± 1.08	5.83 ± 1.40	6.14 ± 1.48
Carrot	7.14 ± 1.59	7.06 ± 1.62	6.91 ± 1.56	5.97 ± 1.40	6.49 ± 1.66

**Table 7:** Soy burger recipe composition.

Ingredient	[g/100 g]	[%]
Textured protein hydration		
Textured soy protein	22	22.00%
Water	22	22.00%
Beet juice powder	0.1	0.10%
Carob	0.2	0.20%
Mass		
Water	33.5	33.50%
Canola oil	4.5	4.50%
Beet juice powder	0.2	0.20%
Carob	0.3	0.30%
Wheat fiber	0.3	0.30%
Pea fiber	2.3	2.30%
Soy protein	4.5	4.50%
Potato starch	1.7	1.70%
Dried onion	2.5	2.50%
Pectin	1.7	1.70%
Xanthan	1.7	1.70%
Beef seasoning	0.9	0.90%
Salt	0.6	0.60%
Dried vegetable ingredient	1	1.00%

## Data Availability

The original contributions presented in this study are included in the article/Supplementary Materials. Further inquiries can be directed to the corresponding authors.

## Supplementary Materials

The following supporting information can be downloaded at: https://www.mdpi.com/article/10.3390/molecules31050912/s1, Table S1: Polyphenol and flavonoid content [mg/100 g] and DPPH Radical Scavenging Activity [%] of samples of dried plant powders.
