# Supplementary material for: Effect of Textured Plant Protein Granulation and Presence of Dried Plant Ingredients on Physicochemical Properties of Soy-Based Burger
Source: Molecules. 2026 Mar 9;31(5):912. doi: 10.3390/molecules31050912 (PMC12985979; doi:10.3390/molecules31050912)
Supplement: Supplementary file 1 [file molecules-31-00912-s001.zip › molecules-4175194-supplementary.pdf]

Table S1. Polyphenol and flavonoid content [mg/100g] and DPPH Radical Scavenging Activity [%] of samples of dried plant powders.

Group	Polyphenols [mg GAE/100 g]	Flavonoids [mg Quercetin/100 g]	DPPH Radical Scavenging Activity [%]
Cardamon	7298.34±73.77	3086.97±16.90	81.17±0.97
Pomegranate	7491.14±35.20	3196.84±38.00	84.15±1.12
Juniper	4803.76±86.77	2828.09±18.61	77.93±0.67
Carrot	3307.32±88.81	2239.48±22.58	69.75±0.84
